# Gut-on-a-Chip for the Analysis of Bacteria–Bacteria Interactions in Gut Microbial Community: What Would Be Needed for Bacterial Co-Culture Study to Explore the Diet–Microbiota Relationship?

**DOI:** 10.3390/nu15051131

**Published:** 2023-02-23

**Authors:** Ki Won Lee, Jin Song Shin, Chan Min Lee, Hea Yeon Han, Yun O, Hye Won Kim, Tae Jin Cho

**Affiliations:** 1 Department of Food and Biotechnology, College of Science and Technology, Korea University, 2511, Sejong-ro, Sejong 30019, Republic of Korea; 2 Department of Food Regulatory Science, College of Science and Technology, Korea University, 2511, Sejong-ro, Sejong 30019, Republic of Korea; 3 Department of Animal Sciences, University of Illinois at Urbana-Champaign, Urbana, IL 61801, USA

**Keywords:** lab-on-a-chip, bacterial co-culture, microbial interaction, synthetic gut microbiome, ecological model, commensal bacteria, probiotics, pathogens, diet–microbiota relationship

## Abstract

Bacterial co-culture studies using synthetic gut microbiomes have reported novel research designs to understand the underlying role of bacterial interaction in the metabolism of dietary resources and community assembly of complex microflora. Since lab-on-a-chip mimicking the gut (hereafter “gut-on-a-chip”) is one of the most advanced platforms for the simulative research regarding the correlation between host health and microbiota, the co-culture of the synthetic bacterial community in gut-on-a-chip is expected to reveal the diet–microbiota relationship. This critical review analyzed recent research on bacterial co-culture with perspectives on the ecological niche of commensals, probiotics, and pathogens to categorize the experimental approaches for diet-mediated management of gut health as the compositional and/or metabolic modulation of the microbiota and the control of pathogens. Meanwhile, the aim of previous research on bacterial culture in gut-on-a-chip has been mainly limited to the maintenance of the viability of host cells. Thus, the integration of study designs established for the co-culture of synthetic gut consortia with various nutritional resources into gut-on-a-chip is expected to reveal bacterial interspecies interactions related to specific dietary patterns. This critical review suggests novel research topics for co-culturing bacterial communities in gut-on-a-chip to realize an ideal experimental platform mimicking a complex intestinal environment.

## 1. Introduction

Gut microbes are directly linked to human health, and the development of food products for gut functionalities has mainly focused on maintaining the dominance of beneficial microorganisms with probiotics and/or prebiotics [1,2,3]. Food components can directly act as nutritional substances or indirectly alter human health through metabolites produced by gut microbiota [4,5]. The interaction between diet and gut microbiota can be represented as the bacterial utilization of dietary materials; thus, the role of gut microbiota in human health should be revealed by examining the bacterial metabolic pattern [6,7].

However, individuals show different health outcomes after consumption of the same food products due to the diverse composition of the gut microflora [8,9]. To develop personalized nutraceuticals designed for diet–host relationships associated with gut microbiota, an in vivo study using model living organisms (e.g., mice, guinea pig) was conducted to validate the results of the comparative analysis on the gut microbiota of individuals according to different dietary interventions (e.g., administration of probiotic strains and controlled feeding based on dietary guidelines) [10,11]. The gnotobiotic mouse model established by the colonization of isolated human gut microbiota in germ-free organisms has been widely used to mimic the environment of the human gut as simulative research regarding the relationship between diet and gut microbiota [12,13]. However, time-resolved experimental measurement (i.e., short-term sampling interval) of data under precisely controlled environmental variables is difficult for in vivo trials owing to ethical or technical issues, whereas in vitro studies allow the high-throughput screening and repetitive tests required to obtain validated data, which can also be used for in silico models [14,15].

To consider the variation in the complex composition of the gut microbiome, the metabolic interactions exhibited during the co-culture of multiple microbial species categorized as commensal, harmful, or beneficial bacteria should be understood [16]. The direct use of diluents of human feces for investigating the reaction of gut commensals according to the diet has been preferred to obtain practical and reliable results because of the limited number of bacterial species organizing the synthetic gut bacterial consortia and the presence of non-culturable bacterial species in the gut microflora [17,18,19]. However, the availability of an in-depth study to reveal the mode of action for the interaction among specific bacterial species of interests and the reproducibility of the experiments highlight the distinct strength of in vitro co-culture of bacterial strains isolated from intestinal tracts [20]. Moreover, in the case of fecal samples as experimental tools for microflora in the intestinal tracts, the distinct differences between mucosal and fecal communities have been regarded as a major limitation [21,22]. Synthetic bacterial communities artificially organized with model strains of gut microflora components have been recognized as alternatives to the culture of fecal samples [23,24]. The growth and metabolic characterization of the members of co-cultured model strains have revealed bacteria–bacteria interactions which are critically involved in the assembly of the gut microbiome and the production and/or consumption of bacterial metabolites [25,26]. In particular, health-promoting strategies based on the interaction of commensal bacteria with probiotics and pathogens can be represented as the induction of metabolisms beneficial for the host and the prevention of infection, respectively [27,28,29]. To explain the activity of complex gut microflora, recent advances in co-culture studies using isolates from the gut have suggested ecological modeling methods [30], and the culturomic-based reconstruction of synthetic gut bacterial communities organized by metagenomic data has supported the identification of bacterial taxa of interest (the strain level) [20].

Lab-on-a-chip as an intestinal organ chip (hereafter “gut-on-a-chip”) is one of the most advanced in vitro experimental platforms for investigating the impact of the interaction between human and bacterial cells [31,32]. Although various types of gut chips have been designed for the cultivation of human cells with bacteria, previous research has mainly reported the growth of a single strain [33,34,35]. Although the survival of fecal samples isolated from human subjects has been reported to validate the ability of gut-on-a-chip culturing of multiple strains simultaneously, application studies using gut consortia providing insights into the bacterial interaction are scarcely reported [36,37]. Thus, handling mixed model strains in gut-on-a-chip with the established research method to examine the interaction occurring in synthetic consortia is expected to broaden our understanding of the role of bacterial community members in the gut.

In this review article, we focus on the application of synthetic bacterial consortia with gut commensal and/or pathogens to evaluate the health effects of the diet using the following strategies: (1) modulation of the composition of microbiota, (2) induction of desirable metabolic pathways from commensal bacteria, and (3) control of pathogenic bacteria. Moreover, major findings from the research cases on the cultivation of bacteria in gut-on-a-chip are analyzed to suggest future perspectives on the culture of multiple bacterial strains (e.g., gut commensals, probiotics, and pathogens) in gut-on-a-chip for studying the cross-talk of not only the bacteria–bacteria but also human cell–bacteria interactions.

## 2. Classification of the Methods for the Singular and Mixed Culture of the Model Strains of Gut Microbiota

**Figure 1:**
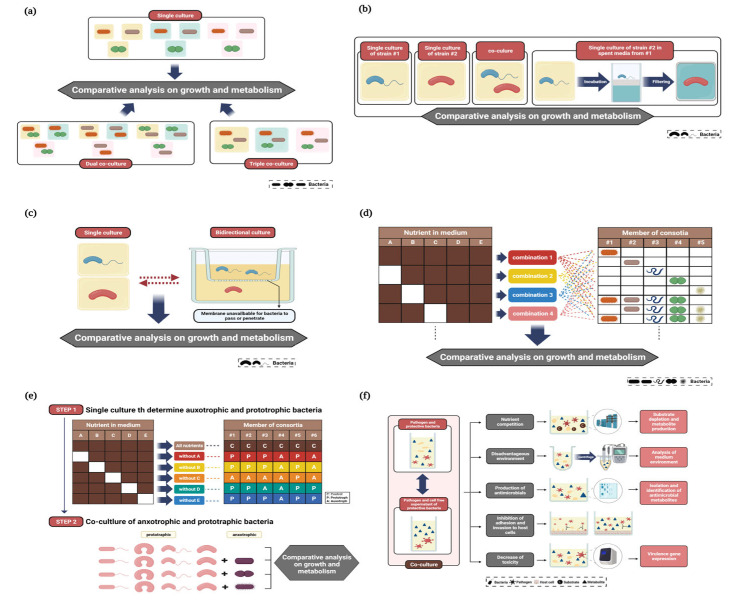
Major methods for singular and mixed culture of model strains of gut microbiota (created with www.biorender.com (accessed on 22 February 2023)). (**a**) Comparative analysis of the singular and co-culture of bacteria under various environments by differentiating the growth determinant factors (**b**) Assessment of the one-way relationship of trophic level by unidirectional culture using spent medium (**c**) Assessment of the two-way relationship of trophic level by bidirectional culture (**d**) Analysis of growth and metabolism pattern in accordance with complex combination of nutrients and members of bacterial consortia (**e**) Induction of syntrophic growth by the cross-feeding during the co-culture of auxotrophics and prototrophics (**f**) Pathogen control strategies based on bacterial interaction.

A schematic diagram of the experiments to culture singular and/or multiple strains that have been reported as members of gut microflora (e.g., commensal bacteria organizing gut microflora, probiotics, and pathogens) is summarized in Figure 1.

As described in Figure 1a, the comparative analysis of the single and co-culture (e.g., dual and triple co-culture) under various environments by differentiating the growth determinant factors, including the nutritional sources and the composition of the media, oxygen concentration, and growth-inhibiting agents (e.g., antibiotics), can provide clues to the direct interaction between bacterial strains. The species-dependent ecological and metabolic features linked to the utilization of dietary polysaccharides allow for the cooperation of bacterial communities to fill gaps in the metabolic pathway for the production of specific metabolites that cannot be obtained from the mono-culture [38]. Optimal microbial consortia can be designed from strategies to induce desirable metabolic interactions among the intestinal microbes with the perspectives of nutritional resource sharing or competition by the direct comparison of mono- and co-culture [39]. The discovery of a cross-feeder that releases the metabolites needed by other bacteria for growth can also be achieved by co-culture experiments exploring various combinations of bacterial strains [40].

To distinguish the impact of bacterial metabolites on another bacterial strains from the bacteria–bacteria interactions caused by the direct contact between test strains, unidirectional culture using spent medium to assess the one-way relationship of trophic level (Figure 1b) or bidirectional culture separating the bacteria through the membrane, which only allows the concurrent diffusion of metabolites during the simultaneous bacterial growth showing a two-way relationship of trophic level, can be adopted (Figure 1c) [41,42].

As an advanced method to elucidate the diverse pattern of growth and metabolism according to the bacterial interaction, the complex combination of not only the members of bacterial consortia but also the nutrients in the media has been applied for co-culture studies (Figure 1d). An examination of the relationship between different mixtures of both nutritional sources and composition of the bacterial community can evaluate the influence of the determinant factors (e.g., supply of the diet and interspecies relationship of competition or cooperation with different resources) on the prioritization of each bacterial species in the community to utilize substrates [43]. Since the use of complex dietary polysaccharides as prebiotics requires multiple steps of degradation and breakdown to be the end-product of the metabolites beneficial for health, previous research regarding the exploration of bacterial consortia with the capacity for the desirable utilization of these polysaccharides has been reported [38,44]. A single species deletion approach from the bacterial consortia has been applied to analyze the niche of each bacterial species with ecological perspectives for estimating the impact of the disappearance of specific bacterial species on the composition of gut microflora, metabolite production, amount of residual substrates, and growth potential [45]. Comparative analysis of the transcription and protein production patterns between single and community cultures also helps to identify co-culture-specific metabolites and evaluate their role in the assembly and/or modulation of the gut microflora [46].

One of the major roles of cross-feeding during co-culture is the induction of syntrophic growth in auxotrophic bacterial strains (Figure 1e). Predetermined auxotrophics according to the lack of the ability for bacterial growth in the absence of specific nutritional substances with the sequential deletion approach can be cross-fed by prototrophics, and finding optimal microbial consortia can provide insights into strategies for the maintenance of the homeostasis of the gut microflora [47]. Screening of the nutrition utilization ability of bacteria grown in defined basal medium supplemented with various nutritional sources can also emphasize the role of cross-feeders during co-culture by syntrophic growth [48].

Previous studies regarding the bacterial interaction of pathogens with gut commensals or probiotics as protective bacteria reported various working principles of protection to prevent infectious intestinal disease and/or intoxication (Figure 1f) [49]. Nutrient competition is identified by substrate use and metabolic patterns among members of the bacterial consortia. A decrease in pH levels along with bacterial growth is a representative disadvantageous environment for pathogens. In the case of antimicrobial substances, treatment with cell-free supernatant (CFS) from protective bacteria can assess the pathogen control activities derived from metabolites released in CFS. The ability of pathogens to adhere to or invade intestinal cells is assessed to estimate the inhibitory effects of protective bacteria (i.e., pre-colonization or competition for colonization) and their metabolites, which can interfere in the pathogen–host interactions. The expression of toxicity-related genes and secretion of toxic substances by pathogens can also be mediated by protective bacteria.

## 3. Recent Advances in Singular and Mixed Culture Studies Using Model Strains of Gut Microbiota

### 3.1. Modulation of Gut Microbiota Composition

Bacterial interactions directly affect the viability and growth of each member of the gut bacterial community through cooperation or competition, which alters the composition and metabolic activity of the gut microflora. Thus, previous relevant research aims to establish strategies that induce desirable changes in community assembly (e.g., dominance of beneficial bacteria and modification of dysbiosis) and metabolic profiles. Key topics of the research area regarding the determinant factors of the composition and metabolism of gut microflora can be categorized as follows (Table 1): (1) discovery of the agents and/or bacterial species supporting syntrophic bacterial growth and (2) prediction of gut bacterial community assembly by metabolic and/or growth modeling.

In the case of syntrophic growth, a comparative analysis of the growth yields between single and co-culture growth in a defined medium with limited resources to bacteria was conducted to establish a cross-feeding strategy for the stable cultivation of bacteria requiring specific nutritional substances. Egan et al. [40] used mucin-containing media providing mucin as the sole carbon source to demonstrate improved viability of *Bifidobacterium breve* supported by the mucin-degrading activity of *Bifidobacterium bifidum*. To understand the modulation of gut commensals according to dietary fiber, the simultaneous consumption of multiple polysaccharides should be considered because of the following changes in the preferential order and the extent to which the substances are utilized by commensals. Liu et al. [43] showed the compositional variation of the bacterial community according to different combinations of co-cultured strains from gut commensals and plant polysaccharides to provide clues for the induction of the dominance of specific bacterial species. To prevent dysbiosis due to the disappearance of specific microflora-organizing bacteria supporting the growth of major commensals linked to human health, a comparative analysis of the changes in growth and metabolic characteristics following the deletion of one bacterial species from the microbial consortia can provide clues for finding the key cross-feeder. Gutiérrez and Garrido [45] co-cultured representative gut microbiomes (five species from *Firmicutes*, seven species from *Bacteroidetes*, one from *Proteobacteria*, and one from *Actinobacteria*) and derived an interaction network based on the correlation matrix of the variables to reveal that the keystone species substantially changed the production of health-functional metabolites and cross-feeding beneficial bacterial species. Demonstrating the growth requirements of beneficial bacteria is crucial to promote their dominance in the gut, especially in cases where their abundance is altered. Previous research has highlighted the importance of the cross-feeder during the mixed culture of auxotrophic and prototrophic strains [51]. Since the syntrophic communities organized with auxotrophic phenotype mutants generated by the gene knockout prototrophic bacterial strain have focused on the role of metabolic cross-feeding as a driver to assemble synthetic microbial consortia [52,53,54], an in-depth understanding of cooperation and competition due to the limited nutritional resources among the variable species of gut commensals is expected to establish a strategy that induces the dominance of beneficial commensals. Soto-Martin et al. [47] analyzed genomic features governing the micronutrient requirements of vitamins at different concentrations from 15 butyrate-producing species commonly detected as commensals in the gut and explored the capacity of prototrophs to share metabolites with other auxotrophic members in the community.

To understand the procedures for the assembly of bacterial members of gut microflora, ecological models for considering the taxonomic complexity of synthetic communities have been developed and applied using either the bottom-up approach (in vitro or in vivo to in silico) or the top-down perspective starting with the system (in silico to in vitro or in vivo) [20,55]. Modeling of the metabolic networks for individual species and bacterial communities by using the data generated from the mono- and co-culture is the typical study design of bottom-up approaches to systems. Petruschke et al. [46] cultivated the simplified human intestinal microbiota (SIHUMIx) members as a single strain to find small proteins (sProteins) expressed only from the co-culture of SIHUMIx and conducted metabolic community modeling with the in silico knockout of the corresponding reactions to evaluate the impact from the absence of those sProteins by analyzing the changes in the metabolite exchange patterns and intestinal community shaping. Genome-guided metabolic modeling applied to members of synthetic bacterial consortia using a bottom-up approach has also identified the major drivers of the structure and function of the community. Weiss et al. [41] developed an integrated theoretical–experimental–modeling framework based on the mono- and pairwise co-culture of Oligo-Mouse-Microbiota (OMM^12^) strains, showing the inhibitory interaction correlated with the substrate depletion profiles among strains from data of growth analysis in spent culture media and pairwise interactions transferred to the community level through the analysis of the co-culture dynamics. Shetty et al. [15] designed Mucin- and Diet-based Minimal Microbiome (MDb-MM) consortia with a culturing system to validate the similarity of natural gut microflora and to decipher the influence of nutrient periodicity (i.e., diverse pattern of dietary intake and starvation) on the bacterial community response by analyzing bacteria–bacteria and bacteria–environment interactions. In terms of top-down perspectives, in vitro experiments of bacterial growth and metabolism can be used for the validation of in silico simulations of modeled metabolisms from gut microbes to analyze the genotype–phenotype relationship and the interaction occurring in the bacterial community. Magnúsdóttir et al. [50] generated the assembly of gut organisms through reconstruction and analysis (AGORA) using genome-scale metabolic reconstructions (GENREs) to predict interspecies interactions and cross-feeding during the co-culture of AGORA members (*Bacteroides caccae* and *Lactobacillus rhamnosus* GG [LGG]) derived by metabolic modeling, which was validated by co-culture experiments.

### 3.2. Induction of the Desirable Metabolic Pathways from Commensal Bacteria

The major goal of controlling metabolisms in gut microflora is to increase the production of health-beneficial metabolites by commensal and/or probiotic bacterial strains in the community [56,57]. To investigate the determinant factors of the production and consumption patterns of metabolites, the interaction between key bacterial species showing the metabolic pathway to produce health-functional agents should be considered [58,59]. Ecological models explaining bacterial interactions and metabolic fluxes within the community have been applied to the dynamic simulation of complex microbiomes [60,61]. Previous research regarding the experimental approach for using co-culture as an inducing factor of desirable metabolisms is summarized in Table 2.

Cross-feeding has been adopted as a strategy to boost substrate fermentation and the production of beneficial metabolites, and thus a comparative analysis on the metabolic characteristics of the combination of the community (i.e., metabolite producer and cross-feeder) and substrate has been conducted. In particular, strain-dependent traits of metabolism highlighted the importance of strain-level mixed culture to find the best consortia in specific nutritional sources [48]. Since the number of bacterial strains during co-culture (e.g., dual, triple, and quad co-culture) affects the metabolic pattern, the validation of the bacterial combination that is feasible for the maintenance of improved metabolite production should be followed after the discovery of the bacterial cooperative relationship between metabolism and health benefits. Thomson et al. [39] characterized the substrate consumption (xylan and inulin) and the production of short-chain fatty acids (SCFAs) according to the types of paired combinations of gut commensals (*Bacteroides dorei*, *Bifidobacterium adolescentis*, *Clostridium symbiosum*, *Escherichia coli*, and *Lactobacillus plantarum*), and the batch culture of microbial consortia organized with all commensals also showed the key bacterial species that contributed to the production of SCFAs [39]. Comprehensive analysis of mono-, bi-, and tri-culture fermentation patterns of commensals and probiotics can broaden our understanding of the use of dietary substances from food products to establish strategies for gut microbiome modulation, because probiotic utilizers may require polysaccharide breakdown products that can be generated by generalist degraders as cross-feeders to produce SCFAs and/or organic acids from specific carbohydrate substances (e.g., galactomannan) [38]. Cross-feeding interaction during the bacterial co-culture can also be found to be mutual, e.g., as found by Rivière et al. The study in [44] showed that metabolites from the utilization of arabinoxylan oligosaccharides (AXOS) by bifidobacterial (*Bifidobacterium longum* subsp. *longum*) and butyrate-producing (*Eubacterium rectale*) strains mutually cross-fed due to the mechanism of bifidogenic and butyrogenic effects. As probiotic bacterial strain utilizing prebiotics are expected to act as cross-feeders by producing metabolites required for the growth of other gut commensals, co-culture studies have also been conducted to assess resource sharing [62,64]. Flux exchange predicted by potential metabolic characteristics from bacterial genome data can be confirmed through unidirectional and bidirectional culture assays to assess contact-independent interactions solely relying on metabolic exchange. Hirmas et al. [42] emphasized the importance of the bidirectional culture method as a tool for the validation of synergistic growth supported by the cross-feeding flux between gut bacteria because the stimulatory effect of increasing the growth of *Lachnoclostridium symbiosum* bidirectionally cultured with *Phocaeicola dorei* could not be observed by unidirectional culture, highlighting the commensal interaction mainly due to the dynamic exchange of metabolites. To overcome the complexity of the gut microbiome, research regarding interspecies metabolic interactions has aimed to create synthetically defined co-culture models that can simulate the gut microbiome by using the minimal combination of bacterial strains. Shetty et al. [63] assembled a Db-MM organized with core bacteria that utilize substrates categorized into trophic levels (complex substrate degrader, simple sugar degrader, and fermentation by-products utilizer), and transcriptome-based analysis of the bacterial relationship (complementary/cooperation or competition) reported active cross-feeding, which supported the co-existence of Db-MM strains and strain-specific activity with respect to carbohydrate utilization via gut metabolic modules.

### 3.3. Control of Pathogens

Since pathogenic microorganisms can directly attack intestinal cells and produce metabolites that cause disorders in the gut, biological intervention strategies using protective bacteria have reported the inhibitory effects of colonization and metabolism along with the growth of pathogens [49]. Previous studies have reported the intervention of pathogens (e.g., colonization resistance and disease tolerance) derived from the interspecies interaction during the co-culture of bacterial strains and established a protective bacterial community to suggest a desirable composition of gut microflora resistant to intestinal infection (Table 3).

#### 3.3.1. Co-Culture of Pathogenic and Protective Bacterial Strains

Competitive interactions between pathogens and protective bacteria can be assessed using solid medium as the growth environment (e.g., agar well diffusion assay) [80], but dynamic exchange of metabolites and direct bacterial-contact-based inhibitory effects should be assessed by liquid co-culture assays. The major mechanisms expected by protective bacteria are as follows: (1) nutrient competition, (2) construction of disadvantageous environment, (3) production of antimicrobial substances, (4) inhibition to adhesion to and invasion of epithelial cells, and (5) a decrease in pathogenic toxicity [81].

Nutrient competition can be assessed by analyzing the consumption of resources followed by the dynamic flux of metabolites when pathogens are inhibited during co-culture with commensals and/or probiotics. Selective inhibition of pathogens in minimally defined media (e.g., M9) supplemented with a carbon source can reveal a competitive correlation with co-cultured bacteria. Osbelt et al. [65] showed colonization resistance by carbohydrate competition between pathogenic (*Klebsiella pneumoniae*) and commensal (*Klebsiella oxytoca*) strains within the genus *Klebsiella*. Sudan et al. [66] confirmed the contact-dependent inhibition of *Bacillus subtilis* against enterotoxic *E. coli* (ETEC) since the inhibitory effect of the co-culture was not observed when CFS of *B. subtilis* was treated with ETEC, and the production of downstream metabolites (indole and kynurenic acid) with the depletion of tryptophan utilized by both bacteria suggested nutrition niche competition as the working mechanism.

Disadvantageous environments, especially due to decreased pH levels originating from bacterial metabolites, can inhibit the growth of acid-susceptible pathogens [68]. The results of the time-kill assay during the bacterial co-culture conducted by Chen et al. [67] described the correlation of the reduced pH level by the organic acid production of *Lactobacillus* spp. with the inactivation pattern of carbapenemase-producing *Enterobacteriaceae*.

The assessment of antimicrobial effects and component analysis of CFS from probiotics and/or commensals can provide information regarding the mode of action of pathogen control supported by bacterial interactions [68,69,70,71]. Kathayat et al. [68] showed the morphological changes of avian pathogenic *E. coli* (APEC) exposed to CFS from probiotics (LGG and *Bifidobacterium lactis* Bb12) and, following isolation of bioactive molecules from CFS, were able to identify antibacterial small peptides effective for APEC. Chen et al. [69] obtained *E. coli* mouse small intestine isolate 1 (EMS1) as a protective bacterium against *Vibrio cholerae* from the mouse gut microbiome by screening commensals with anti-*Vibrio* activity and suggested colibactin as an active substance through genetic analysis. However, the contact-independent inhibitory effect generally analyzed by the bidirectional co-culture of bacterial strains separated by a membrane could not be estimated because of the large size of colibactin that passes through the membrane (10 kDa molecular weight). This phenomenon implies that a reliable analysis cannot be performed when the size of the generated metabolites is larger than the pore size of the membrane during the separated co-culture study (Figure 1C) to verify the effect of secreted metabolites without considering bacterial-contact-derived interactions [69]. Huedo et al. [70] confirmed the inactivation of foodborne pathogens (enteropathogenic *E. coli*, *K. pneumoniae*, *Salmonella enterica* subsp. *enterica* serovar Typhimurium, *Staphylococcus epidermidis*, and *Staphylococcus aureus*) by treatment with antimicrobial substances (e.g., organic acid and bacteriocin) produced from probiotics (*B. longum* and *Pediococcus pentosaceus*) and emphasized the synergistic effects of the simultaneous growth of multiple probiotic strains. Most previous relevant studies isolated antimicrobial substances after co-culture screening of active protective bacteria, whereas the identification of antimicrobials from the culture of protective bacteria can also be conducted prior to co-culture tests with pathogens. Piazentin et al. [71] evaluated the antimicrobial capability of *Enterococcus faecium* producing bacteriocin-like inhibitory substances (BLIS) through co-culture with various pathogenic species (*Listeria monocytogenes*, *S. enterica*, and *S. enterica* serovar Typhimurium) to determine its antibacterial spectrum. Anti-biofilm effects are one of the major roles of protective bacteria in preventing the long-term survival of pathogens in the gut [72,73,74,75,77,82]. Boopathi et al. [72] suggested that bacterial concentration is the determinant factor for producing antimicrobial metabolites by *B. subtilis*, which can inhibit biofilm formation by *Pseudomonas aeruginosa*. Fang et al. [73] reported a species-specific spectrum of the anti-biofilm effects of *DegP* produced by *E. coli* (i.e., biofilm formation of target pathogens (enterohemorrhagic *Escherichia coli*, *S. aureus*, and *S. epidermidis*) was suppressed, except for *P. aeruginosa*).

Pre-colonization or co-culture of protective bacteria (probiotics and commensals) in the gut is expected to prevent tight junction desorption and cytolysis of epithelial cells caused by the adhesion and invasion of pathogens [68,74,76,82,83,84]. Sudan et al. [66] analyzed the transcription profiles of ETEC during the co-culture of *B. subtilis* and suggested a reduction in the adhesion ability of ETEC due to the motility boost through the increased expression of the motility-flagellar gene (*motA*) but decreased expression of genes involved in adhesion (*faeG* and *tnaA*). Probiotics mainly interfere with the pathogen through pre-adhesion to cells, whereas the production of CFS can also prevent host cell adhesion. Kathayat et al. [68] reported the inhibition of pathogen (APEC) attachment to epithelial cells by treatment with CFS from a probiotic strain (*L. rhamnosus*).

Quantitative analyses to measure the degree of toxic substances and subsequent genetic analyses have been conducted to determine the ability of protective bacteria to control the toxigenicity of pathogens [83]. Sudan et al. [66] confirmed inactivation of toxin-related gene expression (*estA* (heat-stable enterotoxin A), *estB* (heat-stable enterotoxin B), and *eltA* (heat-labile enterotoxin A)) by both co-culture and CFS treatment of protective bacteria with pathogens. Mao et al. [77] revealed that *Faecalibacterium prausnitzii* produces the NLRP6 inflammasome, which can induce the release of cytokines and antimicrobial peptides to protect the host from *Candida albicans* by reducing the activity of virulence factors (proteinase, extracellular phospholipase, and hemolysin).

#### 3.3.2. Design of Protective Bacterial Consortia

The protective mechanism of the synthetic gut microbiome has been confirmed by the assessment of the pathogen colonization resistance. Previous researchers adopted bottom-up construction of the bacterial community for the comparative analysis on the protective activities among the various compositions of the community to figure out the optimally desirable organization of the gut microflora resistant against enteropathogenic infections [78,79].

Comparative analysis on the capability of pathogen control among synthetic gut consortia with variable compositions can provide clues for designing desirable protective bacterial communities. Hromada et al. [78] showed diverse bacterial interactions of *Clostridioides difficile* during co-culture with consortia according to the community members (different mixtures of bacteria from two to thirteen strains within the genus *Acteroidetes*, *Firmicutes*, *Actinobacteria*, and *Proteobacteria*) and the analysis on the ecological characterization of gut consortia, which showed marked effects against *C. difficile* and identified the key driver of the community assembly as *Clostridioides hiranonis* through the working mechanism of resource competition. Phenotypic characterization of a single bacterial strain to discover consortia members with distinctly high inhibitory effects on pathogens is expected to suggest an optimized mixture of protective bacteria. Ghimire et al. [79] assessed the direct inhibition and auxotrophic correlation of *C. difficile* with each culturable bacterial strain isolated from human feces using culturomics to select the members to organize the synthetic gut consortia and reported the desirable protective effects during the co-culture of designed consortia with *C. difficile*.

## 4. Current Status and Future Perspectives on the Research Regarding the Interaction of Model Bacterial Strains in Gut-on-a-Chip

### 4.1. Overview of the Bacterial Culture in Gut-on-a-Chip

The major results and implications from the previous experimental cases that reported the culture of bacteria in gut-on-a-chip are summarized in Table 4.

#### 4.1.1. Establishment of the Experimental Platform Mimicking the Gut Environments for Host and Bacterial Cells

Investigation of the determinant factors of bacterial cytotoxicity potential (i.e., factors inducing cell damage and decreasing cell viability) is needed prior to the evaluation of the beneficial activities of bacteria to determine the experimental conditions of host bacterial culture in gut-on-a-chip [87].

Different oxygen requirements between host and bacterial cells are one of the major challenges in the research regarding the diet–microbiota–host relationship, and thus strategies for the control of oxygen concentration in gut-on-a-chip have been reported [37,86,88]. Shah et al. [86] assessed the applicability of the HuMiX model to support the bacterial growth of both facultative (LGG) and obligate anaerobes (*B. caccae*) using a modular microfluidic device perfusing dedicated culture media to create the aerobic conditions required for human cells. The flow of well-oxygenated medium for human cells during incubation under strictly anaerobic conditions for bacteria permitted the control of oxygen gradients in gut-on-a-chip, and the growth of obligate anaerobic bacteria (*Bacteroides fragilis*) was used as an indicator of the anaerobic conditions established in gut-on-a-chip [37]. Shin et al. [88] designed gut-on-a-chip available for the control of oxygen (anoxic–oxic interface chip) to allow the culture of anaerobic bacteria (*Bifidobacterium adolescentis* and *Eubacterium hallii*). Moreover, the mono-culture and dual co-culture both showed stable bacterial growth without damage to intestinal cells in the chip (confirmed by TEER, ZO-1, and mucin-2).

Bacterial cultures have been used to examine inflammatory responses derived from the gut microbiome in inflammation-on-a-chip. A pathomimetic gut-on-a-chip model was developed to explore the mechanism of the onset of intestinal inflammation based on the comprehensive cross-talk not only from epithelial immune cells but also from the intercellular host–microbiome relationship by exploring the role of *E. coli* in the production of cytokines after inflammation induced by treatment with dextran sodium sulfate (DSS) [85].

The operational system of gut-on-a-chip designed for the simulation of the physiological environment of the intestinal tract was reported. A simulation method of the feeding-environment-dependent changes in the composition of the gut bacterial community was established by connecting a continuous gut dynamic simulator (e.g., SHIME) to gut-on-a-chip, highlighting the ability of these integrated culture systems to explore the determinant effects of diet with perspectives on the modulation of gut microbiota [36].

The physiological characteristics of intestinal cells can be affected by the presence of bacteria in gut-on-a-chip [86,89]. Gene expression profiles of Caco-2 cells were affected by the experimental platform (static and continuous culture using transwell and gut-on-a-chip, respectively) and the presence of bacteria co-cultivated in gut-on-a-chip (cultivation of Caco-2 cells with or without VSL#3 in gut-on-a-chip) [89]. However, Shah et al. [86] reported the differences in the gene expression profiles of in vitro gut-on-a-chip cultivation (Caco-2 cell culture with a single bacteria LGG) compared to in vivo tests and assumed the reason for this phenomenon to be the reduced complexity of the model strain (LGG) compared with actual human gut microflora, highlighting the requirement of the bacterial community for accurate simulative analysis of host–microbiota interplay.

#### 4.1.2. Probiotic Bacteria in Gut-on-a-Chip

The ability of bacteria to adhere to host gut cells is one of the major determinants of the health functionalities of probiotics. Marzorati et al. [36] established the gut-on-a-chip-based host–microbiota interaction module that could enhance the colonization capability of a probiotic strain (LGG) on the mucus layer by providing shear forces and microaerophilic conditions.

The interaction between host and bacterial cells in gut-on-a-chip enables the determination of the determinant factors of the activities of probiotics [33,85,86,87]. Shin and Kim [85] emphasized the importance of an intact intestinal barrier to achieve the health effects of probiotics (e.g., intestinal barrier function), which cannot be observed in damaged cells before the administration of probiotics due to the irreversibility of those damages (e.g., impaired tight junction and decreased mucus production). Nelson et al. [33] demonstrated the applicability of the predictive mechanistic model framework established using gut-on-a-chip to recapitulate the in vivo activity of SYN5183 (symbiotic designed to consume phenylalanine in the gut by phenylalanine ammonia lyase) demonstrated from the dose-dependent increase in trans-cinnamic acid (TCA) as a strain-specific biomarker with the flux of bacterial and host metabolites between the gut lumen and blood compartment. This study highlights the continuous flow of substrates in gut-on-a-chip, which represents the dietary intake of food substances, and the subsequent diffusion of the metabolites produced from bacteria between the gut–blood compartment of the chip can provide background data to develop a metabolic model for estimating the activity of probiotics [33]. Shah et al. [86] showed different cellular metabolic responses, including cross-feeding due to host cell–bacteria interplay in the gut and the decrease in inflammatory cytokines screened by immunological markers [interleukin-8 (IL-8) and CCL20] released from epithelial cells, implying the anti-inflammatory effects of *L. rhamnosus*.

The mechanism of the bacterial role in sensing disease-correlated substances (e.g., gut hormone) could also be examined by using gut-on-a-chip through the responsive metabolic patterns (e.g., sensing sensitivity, production of corresponding metabolites, and transfer between gut and blood vessels) and immune actions (e.g., production of cytokines) [87].

#### 4.1.3. Pathogenic Bacteria in Gut-on-a-Chip

Simulating the occurrence of human infectious diseases is the major aim of previous relevant studies regarding the culture of pathogens in gut-on-a-chip [34,89,91]. Gazzaniga et al. [91] showed that the culture of *S. typhimurium* in gut-on-a-chip could induce the following representative responses of host cells against infection: epithelial injury (e.g., disruption of tight junctions, formation of epithelial lesions, and detachment of epithelial cells) and production of chemokines. Kim et al. [89] established a human intestinal model that could show pathogen-dependent cellular responses to infection by introducing enteroinvasive *E. coli* in gut-on-a-chip; however, these responses were not observed after exposure to non-pathogenic *E. coli* or LPS endotoxin. Grassart et al. [34] suggested intestinal microarchitecture with intestinal flow and peristalsis as a key contributor to the recapitulation of in vivo *Shigella* invasion, highlighting the importance of the simulation of the human gut by 3D cell culture and the application of the mechanical forces available in the intestine chip to accurately predict the pathogen localization causing infection. Tovaglieri et al. [35] recapitulated the species-specific responses to bacterial infection by demonstrating more severe epithelial injuries induced by gut-on-a-chip cultivation of human cells with EHEC in the presence of human microbiome metabolites (Hmms) compared with mouse microbiome metabolites (Mmms). Moreover, Hmms could contribute to the increase in EHEC virulence by upregulating the relevant gene expression (bacterial chemotaxis and motility), and a significantly higher effect was observed after treatment with Hmms than with Mmms [35].

#### 4.1.4. Co-Culture of Multiple Microbial Strains in Gut-on-a-Chip

The aims of the microbial co-culture study using gut-on-a-chip can be categorized as follows: (1) assessment of the impact of multiple microorganisms co-existing with epithelial cells, (2) introduction of gut microbiome isolated from human feces, and (3) evaluation of the protective effects of probiotics against intestinal damage caused by pathogenic infection.

Consortium co-culture of LGG with *Bacteroides caccae* in HuMiX induced transcriptional and metabolic changes in human cells compared with the mono-culture of LGG, highlighting the capability of gut-on-a-chip to capture the different responses according to the consortium composition and the importance of introducing a bacterial community to simulate the gut environment [86].

The human gut microbiome obtained from stool samples has been suggested as one of the indicators to evaluate the usability of gut-on-a-chip, maintaining the viability of multiple bacterial strains and intestinal cells in the established experimental platform [36,37]. Since the cytotoxicity of bacteria to human cells has limited the short-term experimental time of the simultaneous culture of cells with the gut microbiota, the HMI module was designed to indirectly expose Caco-2 cells to complex gut microbiota separated by the mucus layer with a semi-permeable layer, allowing the exchange of metabolites. The viability of enterocytes was validated for up to 48 h, which represents the estimated in vivo enterocyte–bacteria contact time in the gut lumen [36]. Jalili-Firoozinezhad et al. [37] showed that gut-on-a-chip could cultivate primary human intestinal epithelium along with direct contact with the gut microbiome isolated from human stool samples, implicating the potential capabilities of gut-on-a-chip for application in the development of personalized therapeutic treatments.

In the case of probiotics–pathogen co-culture, the pre-colonization of probiotics (VSL#3) could enhance the intestinal barrier function and delay the onset of epithelial cell injury in gut-on-a-chip [89]. The microphysiological intestine chip model established by Maurer et al. [90] was also used to demonstrate that the pre-colonization of *L. rhamnosus* as a protective microbe against *C. albicans* infection could limit fungal growth at the luminal side and translocation into the endothelial compartment. Gazzaniga et al. [91] showed that the pre-colonization of *E. faecium* (isolated from the human microbiome) in the mouse intestine chip could prevent the overgrowth of *S. typhimurium* and epithelial injury (e.g., tight junction disruption and epithelial lesions) caused by pathogenic infection, highlighting the potential protective capability of the microbiome.

### 4.2. Strategies to Integrate the Study Design of Bacterial Co-Culture into Gut-on-a-Chip

**Figure 2:**
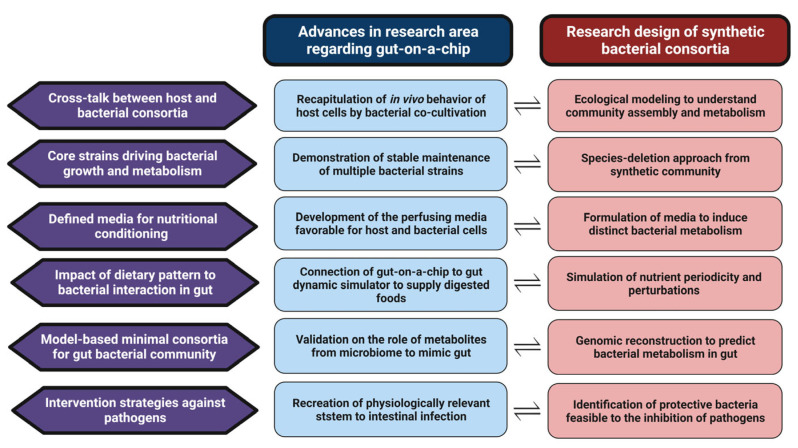
Schematic diagram depicting strategies to integrate study design of bacterial co-culture into gut-on-a-chip (created with www.biorender.com (accessed on 22 February 2023)).

Previous research regarding the culture of bacteria in gut-on-a-chip has highlighted the ability to simulate bacterial growth which can be observed in the mammalian gastrointestinal tract; however, there is a lack of studies on bacterial co-culture with various combinations of bacterial communities and nutritional resources to reveal the bacterial interspecies interactions related to specific dietary patterns. Moreover, experimental cases on the co-culture of multiple bacterial strains in gut-on-a-chip have mainly reported the use of fecal samples, which cannot be applied to the modeling of compositional variations in the microbiome along the intestinal tract (mucosal–luminal axis) [92]. Various community structures of gut microflora can be embodied using synthetic microbial communities organized with biologically relevant bacterial strains, and mathematical models have provided clues for the ecological interactions of community members under controlled nutritional conditions [20]. The following future research perspectives using gut-on-a-chip for co-culturing synthetic bacterial communities are summarized in Figure 2: (1) exploration of cross-talk between host and synthetic gut consortia, (2) discovery of core strains driving bacterial growth and metabolism, (3) development of defined media for nutritional conditioning, (4) impact assessment of dietary pattern on bacterial interactions in the gut, (5) establishment of modeling-based minimal gut bacterial consortia, and (6) improvement of intervention methods against pathogens.

A co-culture study designed for the analysis of bacterial interactions involved in the growth, metabolism, and inactivation of members in the synthetic gut bacterial community is expected to reveal not only the bacteria–bacteria but also the bacteria–host relationship by adopting gut-on-a-chip as a culture platform. The cross-talk between host and gut microflora has also been reported by the cultivation of human cells with bacteria in gut-on-a-chip [85], highlighting its potential to affect bacterial behaviors, which can result in changes in the assembly and metabolism of the microbiome community. Shah et al. [86] revealed that the difference in the transcriptomic profiles derived from epithelial cells cultivated with a single probiotic bacterial strain in gut-on-a-chip compared with in vivo gene expression data from human subjects is likely due to the absence of the complexity from co-cultured bacteria, which emphasizes the importance of community-level bacterial culture to investigate host–microflora interactions in intestinal tracts. Since bacterial cross-talk in the synthetic gut community determines the abundance of community members, ecological modeling can be used to modulate community assembly to induce the dominance of beneficial bacteria, and the estimated health outcome of this modulatory strategy is expected using gut-on-a-chip [41,42,46,63]. As shown from the bidirectional co-culture assay to analyze the contact-independent bacterial interaction by the dynamic exchange of metabolites [42], gut-on-a-chip can also be a useful tool for understanding the overall metabolic flux of host and bacterial cells.

Analysis of the co-culture of multiple bacterial strains enables identification of the core strain driving community assembly and metabolism of interest [40,43,47]. Co-culture in gut-on-a-chip has demonstrated the viability of the bacterial community (e.g., probiotic mixture and fecal samples) [86,88] or the protective effect of pre-colonized probiotic strains against subsequent fungal infection [33]; however, there is a lack of in-depth analysis on the niche of cultured strains from the perspective of bacterial interactions. After the establishment of the model bacterial communities representing human gut microbiota variation in gut-on-a-chip, the species deletion approach from each community is expected to broaden our understanding of the role of members in the consortia [45]. Moreover, host-derived carbohydrates (e.g., mucin glycoprotein) were reported to be degradable by a limited number of commensal bacteria owing to the complex and variable structure of the oligosaccharide chains [40], which implies that evaluating the bacterial metabolic activity to degrade those carbohydrates and to cross-feed other commensals in gut-on-a-chip can identify the key bacterial strains expected to be active in the intestinal tract. The ability to utilize the substrate and relevant metabolic activity is generally reported to be strain-dependent within the same bacterial species [93,94]; thus, the discovery of key drivers for the overall metabolic profile of the gut microflora should be conducted by strain-level experiments.

The development of a culture medium that satisfies the requirements of both gut-on-a-chip and synthetic bacterial communities should be regarded as a major goal to establish a reliable gut simulation system for host and bacterial cells. A defined medium to create an environment for the observation of specific bacterial interactions among the synthetic gut consortia according to their nutritional requirements is the success criterion to reveal the mode of action in estimating food–microflora relationships in the gut [43,47]. Recent progress in culturomics to integrate genomic and metabolomic data for the development of a novel medium has resulted in the growth of commensal bacterial species previously reported as non-culturable [95,96]. In the case of gut-on-a-chip, the formulation of perfusion medium reproducing the environments of the intestinal tract in microfluidic channels should be favorable for the viability of human cells due to the potential cytotoxicity of inoculated bacteria [86,87]. Anoxic or oxic culture medium flowing into gut-on-a-chip can also control the oxygen gradient for culturing an obligate anaerobic gut microbiome with epithelial cells; thus, the role of oxygen supplementation should also be considered when designing the medium [37,88]. The applicability of the medium developed for synthetic gut community in gut-on-a-chip followed by the modification of the medium composition optimized for the maintenance of the bacterial culture with cells and/or the expected function of the medium (e.g., the creation of an oxygen gradient) should be followed.

To assess the impact of actual dietary patterns using gut-on-a-chip, media are fed comprising food components and culturing environments are established that enable the long-term growth and/or survival of both host and bacterial cells for considering chronic dietary supplementation of food products. The establishment of a feeding system to simulate actual food consumption habits, including nutrient periodicity and perturbations (e.g., addition or removal of bacterial species in the community during the culture, increase or decrease in dietary intake, changes in the mixture of the types of carbohydrates, removal of metabolites, and nutrient starvation), is also needed to understand the diet–microflora relationship [15]. Although supplementation of the perfusing medium in microfluidic channels of gut-on-a-chip can be controlled, most previous studies used medium to optimize the maintenance of human cell and bacterial viability [34,35,36,37], while Marzorati et al. [36] combined the use of a gut-on-a-chip module connected to the gut dynamic simulator (SHIME) for the supply of dietary substances digested in human intestinal tracts. The introduction of a synthetic gut microbial community into this system is expected to recreate an in vitro experimental platform relevant for an in vivo diet–host–microbiota interaction. Microfluidic intestinal cell model devices have also been designed to analyze the fate of food components (e.g., dietary nutrients and bioactive compounds) from consumption to transportation or absorption [97,98], and co-cultivation with gut commensals is expected to provide clues to understand bacterial responses during dynamic digestive processes. Nutrichip is designed to mimic the activation of immune cells during the passage of nutrients through the gastrointestinal tract and can also be adopted as the experimental platform for diet-mediated alteration of the behavior of commensals by the cultivation of synthetic gut microflora [99,100].

Bacterial communities cultured in gut-on-a-chip were mainly fecal microbiota [36,37] or a mixture of probiotics [85,89]; thus, validating the applicability of previously established synthetic communities (e.g., SIHUMIx, OMM12, MDb-MM, and AGORA) should be a high priority [15,41,46,50]. Bacterial interactions, especially for cross-feeding, have been reported to support the co-existence of synthetic culture members, and an ecological model that enables the prediction of the alteration of overall metabolic profiles due to the changes in the experimental platform in gut-on-a-chip is also needed to modify the composition of the synthetic gut microbiota optimized for culturing in gut-on-a-chip [63]. Crude metabolites produced by the human microbiome were suggested as the determinant factor to recapitulate the species-specific infection in gut-on-a-chip [35], and the use of a synthetic community is expected to identify the key metabolite for accurately simulating an infection model.

The major objectives of most research regarding the culture of pathogens in gut-on-a-chip are the recreation of the in vitro experimental system physiologically relevant to the in vivo-like infection, and the overall results reported the determinant factors of bacterial pathogenicity with the perspectives of the operational conditions (e.g., structure of the intestinal microenvironment and application of mechanical forces) [34,35,89]. Changes in the viability and virulence of pathogenic bacteria by optimizing environmental conditions in gut-on-a-chip will also affect the susceptibility or resistance of those pathogens against inhibitory effects derived from the direct (e.g., resource competition) or indirect (e.g., production of antibacterial metabolites) interactions during co-culture with probiotics or gut commensals. Since the intervention strategies for pathogens in gut-on-a-chip mainly rely on the pre-colonization of probiotics or commensals without considering the dynamic bacterial interaction [89,90,91], future studies investigating the role of protective microbiota as both dominant bacteria colonized on the epithelial cells and the biocontrol agents should be performed.

## 5. Conclusions

Based on critical analysis of previous case studies, we suggested the following key topics for future research regarding bacterial interaction in gut-on-a-chip: (1) host–bacteria cross-talk, (2) core strains driving growth and metabolism of gut microflora, (3) defined media for nutritional conditioning, (4) impact of the dietary pattern to bacterial interaction, (5) modeling-based minimal gut bacterial consortia, and (6) intervention methods against pathogens. The development of a novel research design using gut-on-a-chip with the experimental approaches of bacterial co-culture studies to discover under-recognized functionalities of food substances is needed to investigate metabolic interactions in the gut microbiota affected by dietary patterns. Organizing synthetic gut microflora with variable bacterial species and diversifying these consortia models according to the distinct composition of individual gut microbiomes should provide evidence for developing personalized food products and nutraceuticals.

## Figures and Tables

**Table 1:** Strategies modulating gut microbiota composition based on bacterial co-culture to discover bacterial species supporting the syntrophic bacterial growth and to predict gut bacterial community assembly by metabolic and/or growth modeling.

Key Nutritional Source	Composition of Microbial Community ^a^	Major Implications	Reference
Mucin	*Bifidobacterium bifidum*, *Bifidobacterium breve*	Cross-feeding of mucin-degrading *B. bifidum* to enable the growth of *B. breve* in a mucin-based medium	[40]
Mixture of plant polysaccharides (arabinoxylan, xyloglucan, β-glucan, pectin)	*Bacteroides ovatus*, *Bifidobacterium longum* subspecies *longum*, *Megasphaera elsdenii*, *Ruminococcus gnavus*, *Veillonella parvula*	Diversification of the priority for the use of dietary fiber to produce short-chain fatty acids according to the different combinations of both the polysaccharides and polysaccharide-degrading bacteria	[43]
Inulin	*Bacteroides cellulosilyticus*, *Bacteroides dorei*, *Bacteroides finegoldii*, *Bacteroides fragilis*, *B. ovatus*, *Bacteroides thetaiotaomicron*, *Bacteroides vulgatus*, *Bifidobacterium adolescentis*, *Escherichia coli*, *Flavonifractor plautii*, *Lachnoclostridium clostridioforme*, *Lachnoclostridium symbiosum*, *Lactobacillus plantarum*, *R. gnavus*,	Modelling the impact of species deletion from the gut commensal consortia during the community co-culture to the relative growth of dominant species and the production of metabolites	[45]
Vitamin	*Anaerobutyricum hallii*, *Anaerostipes caccae*, *Anaerostipes hadrus*, *Clostridium* sp., *Coprococcus cactus*, *Coprococcus* sp., *Eubacterium rectale*, *Faecalibacterium prausnitzii*, *Roseburia faecis*, *Roseburia intestinalis*, *Roseburia inulinivorans*, *Subdoligranulum variabile*	Categorization of butyrate-producing bacterial species into prototrophic or auxotrophic strains according to the supplementation of the vitamins and the designation of the bacterial community of auxotrophs which can be cross-fed by added prototrophs	[47]
Complex intestinal medium	*A. caccae*, *B. thetaiotaomicron*, *B. longum*, *Blautia producta*, *Clostridium butyricum*, *Clostridium ramosum*, *E. coli*, *L. plantarum*	Identification of novel proteins produced during only the co-culture of SIHUMIx community (i.e., not produced by single culture) and the evaluation of the role of those proteins to organize the bacterial communities	[46]
AF medium ^b^	*Acutalibacter muris*, *Akkermansia muciniphila*, *Bacteroides caecimuris*, *Bifidobacterium animalis*, *Blautia coccoides*, *Clostridium innocuum*, *F. plautii*, *Enterocloster clostridioformis*, *Enterococcus faecalis*, *Limosilactobacillus reuteri*, *Muribaculum intestinale*, *Turicimonas muris*	Generation of the metabolic models of synthetic bacterial consortia (Oligo-Mouse-Microbiota, OMM^12^) from the experiments of directional bacterial interaction using spent media and the comparative analysis on mono-culture with pairwise co-culture to identify key driver of the community structure and function	[41]
Medium composed in this study ^c^	*A. muciniphila*, *Agathobacter rectalis*, *Anaerobutyricum soehngenii*, *B. ovatus*, *Bacteroides xylanisolvens*, *B. adolescentis*, *Blautia hydrogenotrophica*, *Blautia obeum*, *Collinsella aerofaciens*, *Coprococcus catus*, *Eubacterium sireaum*, *F. prausnitzii*, *F. plautii*, *Roseburia intestinalis*, *Ruminococcus bromii*, *S. variabile*	Evaluation of the synthetic minimal microbiome (Mucin- and Diet-based Minimal Microbiome; MDb-MM) regarding the exhibition of the ecological and metabolic features of natural gut microflora through the compositional, transcriptional, and metabolic analysis using community co-culture under the environment simulating diet intake and perturbation	[15]
DMEM 6429 supplemented with vitamin K and hemin (with or without arabinogalactan)	*Bacteroides caccae*, *Lactobacillus rhamnosus*	Validation of the predictive function of in silico metabolic models based on the genome-scale metabolic reconstructions for human gut microbes (assembly of gut organisms through reconstruction and analysis, AGORA) by using experimental results of cross-feeding from bacterial co-culture	[50]

^a^ Microbial community describes the target for the co-culture of more than two microbial strains. ^b^ Modified AF medium was also used for the experiments to test the impact of specific substrates on the composition of the community of bacterial consortia. ^c^ Composition of the medium was newly designed for this study.

**Table 2:** Inducing factors of desirable metabolic pathways from commensals co-cultured with community members of gut microflora.

Key Nutritional Source	Composition of Microbial Community ^a^	Major Implications	Reference
Glucose, starch, inulin, fructooligosaccharides	*Bifidobacterium adolescentis*, *Faecalibacterium prausnitzii*	Suggestion of the combination of the butyrate-producing bacterial strain with the cross-feeder and supportive substrate (carbon source) to enhance the formation of beneficial short-chain fatty acids	[48]
Inulin, xylan	*Bacteroides dorei*, *B. adolescentis*, *Clostridium symbiosum*, *Escherichia coli*, *Lactobacillus plantarum*	Comparative analysis of carbon source utilization and growth pattern (synergistic or negative interaction) according to the paired combination (i.e., dual culture) or microbial consortia organized with gut commensals	[39]
Galactomannan substrates (guar gum, fenugreek gum, locust bean gum, copra meal, PHGG), β-mannooligosaccharide mixture (from guar gum and copra meal)	*Bacteroides ovatus*, *B. adolescentis*, *Lactiplantibacillus plantarum*	Demonstration of the resource sharing of galactomannan during the bacterial co-culture (dual and triple) by the cross-feeding from *B. ovatus* breaking down the polysaccharides to *L. plantarum* and *B. adolescentis* producing short-chain fatty acids and organic acids	[38]
Arabinoxylan oligosaccharides (AXOS)	*Bifidobacterium longum*, *Eubacterium rectale*	Demonstration of the mutual cross-feeding during the co-culture of bacteria for the utilization of AXOS showing butyrogenic and bifidogenic effects by the provision of concomitant metabolite production of acetate-to-butyrate producer (*B. longum*) and xylose from the AXOS substrate to bifidobacterial strain (*E. rectale*), respectively	[44]
Resistant starch (RS)	*Bacteroides thetaiotaomicron*, *Bifidobacterium aderceptis*	Characterization of the degradation of RS by the probiotic strain (*B. aderceptis*) which can cross-feed other gut bacterium (*B. thetaiotaomicron*) by sharing the reducing sugars generated from RS	[62]
Inulin, xylan	*Lachnoclostridium symbiosum*, *Phocaeicola dorei*	Validation of the synergistic growth according to the cross-feeding as the commensalism interaction observed from the bidirectional culture between *L. Symbiosum* and *P. dorei*	[42]
mixture of dietary fibres (pectin, inulin, xylan, cellobiose, starch)	*Agathobacter rectalis*, *Akkermansia muciniphila*, *Anaerobutyricum soehngenii*, *B. ovatus*, *Bacteroides xylanisolvens*, *B. adolescentis*, *Blautia hydrogenotrophica*, *Blautia obeum*, *Collinsella aerofaciens*, *Coprococcus catus*, *Eubacterium siraeum*, *Faecalibacterium prausnitzii*, *Flavonifractor plautii*, *Roseburia intestinalis*, *Ruminococcus bromii*, *Subdoligranulum variabile*,	Assembly of Diet-based Minimal Microbiome (Db-MM) to predict the interspecies correlation among the members of Db-MM with the perspectives to the metabolic niches and trophic roles in gut	[63]

^a^ Microbial community describes the target for the co-culture of more than two microbial strains.

**Table 3:** Pathogen control strategies established from studies regarding the co-culture of pathogens with protective bacteria.

Composition of Microbial Community ^a^		Major Implications	Reference
Target Pathogen	Co-Culturing Bacteria		
*Klebsiella pneumoniae*	*Klebsiella oxytoca*	Demonstration of the nutrient competition in minimally defined media (M9) supplemented with carbon source shared by both pathogen and co-cultured bacteria	[65]
Enterotoxic *Escherichia coli*, *Salmonella typhimurium*, methicillin-resistant *Staphylococcus aureus*	*Bacillus subtilis*	Demonstration of the nutrient competition (based on the production of downstream along with the depletion of tryptophan shared by both pathogen and co-cultured bacteria) and the bacteria-mediated inhibition of host cell adhesion of pathogens	[66]
Carbapenemase-producing *Enterobacteriaceae*	*Lactobacillus piracies*, *Lactobacillus plantarum*, *Lactobacillus rhamnosus*	Construction of disadvantageous environment for pathogens by the decrease in pH level due to the production of acidic metabolites by probiotics	[67]
Avian pathogenic *E. coli*	*Bifidobacterium lactis*, *Lacticaseibacillus rhamnosus*	Validation on the production of antimicrobial substance by the isolation of active substances (small peptides) and the inhibition of pathogen adhesion to epithelial cells as the mechanism of protective bacteria	[68]
*Vibrio cholerae*, *Vibrio fischeri*, *Vibrio fluvialis*, *Vibrio harveyi*	*E. coli* (isolated from mouse small intestine)	Validation of the production of colibactin as an antimicrobial substance of co-cultured bacteria with anti-*Vibrio* activity through both the phenotypic and genetic analysis	[69]
Enteropathogenic *E. coli*, *K. pneumoniae*, *S. typhimurium*, *S. aureus*, *Staphylococcus epidermidis*	*Bifidobacterium longum*, *Pediococcus pentosaceus*	Suggestion of the combination of probiotic strains (*B. longum* and *P. pentosaceus*) to achieve the synergistic effect for the production of antimicrobial substances	[70]
*Listeria monocytogenes*, *Salmonella enterica*	*Enterococcus faecium*	Co-culture-based assessment of the antibacterial spectrum against pathogens after the identification of *Enterococcus faecium* available for the production of bacteriocin-like inhibitory substance (BLIS)	[71]
*Pseudomonas aeruginosa*	*B. subtilis*	Demonstration of anti-biofilm formation effects from antimicrobial substance produced by the culture of high density of *B. subtilis*	[72]
Enterohemorrhagic *E. coli* (EHEC), *P. aeruginosa*, *S. aureus*, *S. epidermidis*	*E. coli* Nissle (EcN)	Demonstration of species-specific anti-biofilm effects (effective for EHEC, *S. aureus*, and *S. epidermidis* but not effective for *P. aeruginosa*) of protein *DegP* secreted from EcN	[73]
EHEC, *S. enterica* serovar Typhimurium	*Lactobacillus casei*, *L. rhamnosus*	Analysis of the various pathogen control mechanisms of probiotics including the production of antimicrobial substance, the inhibition of host cell adhesion and invasion of pathogens, and the decrease in pathogen cytotoxicity	[74]
*Salmonella paratyphi*	*L. casei*, *L. plantarum*	Demonstration of the production of antimicrobial substance to inhibit pathogen biofilm formation	[75]
*Salmonella enteritidis*, *Salmonella infantis*, *Slamonella kentucky*	*Ligilactobacillus salivarius*	Assessment of the inhibitory effects against the adhesion of pathogens to cell by the co-culture with *L. salivarius*	[76]
*Candida albicans*	*Faecalibacterium prausnitzii*	Suggestion of the host protection mechanism of *F. prausnitzii* by reducing the activity of virulence factors of *C. albicans* through the production of NLRP6 inflammasome, cytokines, and antimicrobial peptides	[77]
*Clostridioides difficile*	*Bacteroides ovatus*, *Bacteroides thetaiotaomicron*, *Bacteroides uniformis*, *Bacteroides vulgatus*, *Blautia hydrogenotrophica*, *Clostridium hiranonis*, *Clostridium scindens*, *Collinsella aerofaciens*, *Desulfovibrio piger*, *Eggerthella lenta*, *Euvacterium rectale*, *F. prausnitzii*, *Prevotella copri*	Demonstration of the principles for the diverse capability of the pathogen control among the synthetic gut consortia according to the various compositions	[78]
*Clostridiodies difficile*	*Bifidobacterium bifidum*, *Bacillus licheniformis*, *Bacteroides eggerthii*, *Bacteroides finegoldii*, *B. vulgatus*, *Blautia wexlerae*, *Clostridium nexile*, *Clostridium* sp., *Drancourtella massiliensis*, *Eubacterium eligens*, *Lactobacillus rogosae*, *Megasphaera indica*, *Parabacteroides merdae*, *Prevotella copri*, *Sellimonas intestinalis*	Suggestion of optimized mixture of protective bacterial members selected by the phenotypic characterization of bacterial strains isolated from human feces to assess the inhibitory effects against *C. difficile* (direct inhibition and auxotrophical correlationship)	[79]

^a^ Bacterial community describes the target for the co-culture of more than two bacterial strains.

**Table 4:** Methods and implications from the results of bacterial culture in the gut-on-a-chip as an organ simulation platform.

Category	Microorganism	Cell	Methods	Major Implications	Reference
Host Microbiota Interaction (HMI) module	*Lactobacillus rhamnosus*	Caco-2 cell	Circulation of bacterial culture through the upper mucus layer (1.5 h)	➢ Demonstration of the colonization of *L. rhamnosus* adhered on the mucus layer in chip	[36]
	fecal microbiota	Caco-2 cell	Introduction of complex microbial community from a SHIME reactor in the upper mucus layer	➢ Maintained viability of cells after 48 h of culture with complex gut microbial community from feces	
				➢ Changes in the production of bacterial metabolites which can affect the immune reaction of cells and the composition of the bacterial community according to the environmental condition controlled by SHIME system	
Gut-on-a-chip (Human emulation system)	SYN5183 (Engineered *Escherichia coli* Nissle)	Caco-2 cell + HT-29 cell (4:1 ratio) for gut compartment and human microvascular endothelial cells (HMVECs) for blood compartment	Introduction of SYN5183 diluted in experimental gut medium to the gut compartment followed by the 1 h static incubation for the settlement of bacteria (single dose) or the 12 h continuous dosing of SYN5183 (continuous administration) in the presence of perfusing medium with phenylalanine	➢ Construction of the mechanistic models of the reduction activity against the blood phenylalanine accumulation according to the dosages of synthetic biotic strain (SYN5183)	[33]
Gut inflammation-on-a-chip	Nonpathogenic *Escherichia coli* (GFP-labeled)	Caco-2 cell, peripheral blood mononuclear cells (PBMCs)	Introduction of *E. coli* flowed into luminal microchannel of gut-on-a-chip simulating the inflammatory response of cells treated with or without dextran sodium sulfate (DSS)	➢ Development of the pathomimetic chip model of DSS-induced inflammation through recapitulating the elevation of oxidative stress and inflammatory responses of DSS-treated immune and epithelial cells by the exposure to *E. coli* cells	[85]
	Mixture of probiotics (*Bifidobacterium breve*, *Bifidobacterium longum*, *Bifidobacterium infantis*, *Lactobacillus acidophilus*, *Lactobacillus plantarum*, *Lactobacillus casei*, *Lactobacillus delbrueckii* subspecies *bulgaricus*, *Streptococcus salivarius* subspecies *thermophilus*)		Introduction of probiotics mixture to the luminal microchannel followed by the static incubation 1–2 h for the settlement of bacteria prior to resuming the circulation of the culture medium in gut-on-a-chip simulating the inflammatory response of cells treated with or without DSS	➢ Demonstration of the intact intestinal barrier as a key pre-requisite to elicit the beneficial probiotic activities by the comparison of the administration of probiotic bacteria before (pre-treatment) and after (post-treatment) DSS-induced epithelial damage	
Modular microfluidics-based human–microbial co-culture model (HuMiX)	*Bacteroides caccae*, *L. rhamnosus*	Caco-2 cell, CCD-18Co cell, CD4+ T cell	Inoculation of bacteria (*B. caccae* and/or *L. rhamnosus*) in microbial microchamber separated from cell microchamber by nanoporous membrane and following exposure to the flow of the perfusing anoxic DMEM medium	➢ Confirmation of no apparent cytotoxic effects (evaluated by live–dead staining and fluorescence microscopy) to various types of cells (Caco-2 cell, CCD-18Co, and CD4 + T cell) during the culture with single or dual co-culture of facultative and obligate anaerobic bacteria ➢ Demonstration of the function for simulating oxygen concentrations in human intestinal tissues by the simultaneous perfusion of oxic and anoxic media through microchamber during the co-culture of *L. rhamnosus* + *B. caccae* with Caco-2 cells	[86]
	*L. rhamnosus*	Caco-2 cell		➢ Analysis of cross-talk between Caco-2 cell and *L. rhamnosus* based on the different metabolic activities compared with mono-culture of *L. rhamnosus* ➢ Identification of disparate gene expression from cells cultured with *L. rhamnosus* in chip (known as responsive to *L. rhamnosus*; *elf3*, *cdk9*, *gadd45b*, *pilrb*) compared with in vivo test to human subjects ➢ Confirmation of anti-inflammatory effect of *L. rhamnosus* cultured with Caco-2 cells	
Gut-on-a-chip (Human emulation system)	ECN and SYN (wild type and engineered *E. coli* Nissle, respectively)	Caco-2 cell + HT-29 cell (4:1 ratio) for gut compartment and human microvascular endothelial cells (HMVECs) for blood compartment	Introduction of bacteria (ECN or SYN) to the gut compartment followed by 30 min static incubation for the settlement of bacteria in the presence of perfusing medium with L-tryptophan and cortisol	➢ Determination of the applicability of symbiotic bacterial strain by confirming the absence of the bacterial invasion or translocation into the blood compartment despite the potential of bacterial cytotoxicity ➢ Demonstration of the cortisol-sensing effects of SYN by the bioconversion of tryptophan to tryptamine	[87]
Gut-on-a-chip (Human emulation system)	*Shigella flexneri*	Caco-2 cell	Introduction *of S. flexneri* to the upper channel of the epithelial cells seeded on the central channel (stretchable porous membrane) by 30 min of microfluidic flow (400 μL/h) to expose bacteria over the full surface of the channel	➢ Assessment of the advantages of pathogen infection model using intestine chip feasible for the 3D colonic epithelium culture and the exposure of mechanistic forces for the reliable simulation of *Shigella* infection (polarized) and colonization	[34]
Human primary colon chip	Enterohemorrhagic *E. coli* (EHEC; serotype O157:H7)	Human intestinal microvascular endothelial cells (HIMECs)	Introduction of EHEC for the attachment on the apical lumen cell followed by 3 h static incubation for the settlement of bacteria prior to resuming the circulation of perfusing medium supplemented with human microbiome metabolites (Hmms) or mouse microbiome metabolites (Mmms)	➢ Recapitulation of the species-specific tolerance against pathogens from the higher sensitivity to EHEC infection (loss of epithelial cells) exposed to Hmms compared to Mmms ➢ Demonstration of the cytokine release (pro- and anti-inflammatory) induced by EHEC infection similar to in vivo during the cultivation of human cells in the presence of Hmms ➢ Evaluation of higher stimulatory effects of Hmms compared to Mmms from the increase in the bacterial chemotaxis and motility which contribute to the increase in the virulence of EHEC	[35]
Anoxic-oxic interface (AOI) chip	*Bifidobacterium adolescentis*, *Eubacterium hallii*	Caco-2 cell	Introduction of bacteria (*Bifidobacterium adolescentis* or *ubacterium hallii*) to microbial microchannels (upper channels) for the attachment on the apical epithelial surface followed by the 1 h static incubation for the settlement of bacteria prior to resuming the circulation of anoxic medium in AOI chip	➢ Demonstration of the function of AOI chip to control oxygen supplemented to bacterial or human cells for the survival of representative obligate anaerobic bacteria by the prevention of the damage derived under oxic conditions	[88]
Primary human intestine chip	*Bacteroides fragilis*	Caco-2 cell, HIMECs	Introduction of *B. fragilis* to the apical side of chips followed by 30 min static incubation for the settlement of bacteria	➢ Establishment of stable anaerobic conditions and oxygen supplementation system favorable for obligate anaerobic bacteria and human cells, respectively	[37]
	fresh human stool specimens	primary human intestinal epithelium (epithelial cells isolated from organoids derived from human ileum)	Introduction of diluted microbiota stock to the apical side of chips followed by 30 min static incubation for the settlement of bacteria	➢ Cultivation of primary human intestinal epithelium in direct contact with human gut microbiome without compromising the barrier function and structure of epithelial cells	
Gut-on-a-chip	VSL#3 (*B. breve*, *B. infantis*, *B. longum*, *L. acidophilus*, *Lactobacillus paracasei*, *L. plantarum*)	Caco-2 cell, PBMCs	Introduction of bacteria (VSL#3, GFP-EC, EIEC) to the upper microchannel followed by 1.5 h static incubation for the settlement of bacteria	➢ Demonstration of different gene expression profiles according to the experimental platform (Transwell or gut-on-a-chip) or the presence of bacteria (cultivation of Caco-2 cell with or without VSL#3 in gut-on-a-chip)	[89]
	green fluorescent protein-labeled *E. coli* (GFP-EC),enteroinvasive *E. coli* (EIEC)			➢ Reconstitution of the human intestinal model mimicking inflammation and injury distinctly derived by the infection only from the exposure to pathogen (EIEC), not from the exposure to non-pathogenic *E. coli* or LPS endotoxin isolated from pathogenic *E. coli*	
	VSL#3 (*B. breve*, *B. infantis*, *B. longum*, *L. acidophilus*, *L. paracasei*, *L. plantarum*), EIEC			➢ Evaluation of the in vivo functionality of anti-inflammatory probiotics (VSL#3) to delay the onset of intestinal injury caused by pathogenic infection	
Intestine-on-chip model	*Candida albicans*, *L. rhamnosus*	HUVECs, Caco-2 cells, PBMCs, primary macrophages	Introduction of *L. rhamnosus* to epithelial cell layer (pre-colonization) followed by 1.5 h static incubation for the settlement of bacteria and subsequent introduction of *C. albicans*	➢ Evaluation of the effects of pre-colonization of *L. rhamnosus* on the reduction in tissue damage and translocation of *C. albicans* in the intestinal lumen model and the improvement on the viability of epithelial cell layer	[90]
Mouse intestine chip	*S. typhimurium*	epithelial cells (isolated from duodenal, jejunal, ileal, or colon organoids)	Introduction of *S. typhimurium* to the apical channel followed by 30 min static incubation for the settlement of bacteria	➢ Recapitulation of epithelial injury and chemokine production induced by *S. typhimurium* infection	[91]
	Human microbiome (Hmb), mouse microbiome (Mmb)		Introduction of Hmb or Mmb to the apical channel followed by 30 min static incubation for the settlement of bacteria	➢ Demonstration of the different compositions of microbiome cultured in gut-on-a-chip between Hmb and Mmb as the domination of *Enterococcus* genus and the variation in abundances of multiple genera, respectively	
	*E. faecium* (isolated from Hmb stock), *S. typhimurium*		Perfusion of *E. faecium* through the apical channel for 16 h before the introduction of *S. typhimurium*	➢ Validation of the inhibitory effects of pre-colonized *E. faecium* as protective bacteria against *S. typhimurium*	

## Data Availability

Not applicable.

## References

[1] Ali M.A., Kamal M.M., Rahman M.H., Siddiqui M.N., Haque M.A., Saha K.K., Rahman M.A. (2022). Functional dairy products as a source of bioactive peptides and probiotics: Current trends and future prospectives. J. Food Sci. Technol..

[2] Lelia P.O., Suharoschi R. (2022). Emerging food processing technologies: Probiotics and prebiotics. Nutraceutical and Functional Food Components.

[3] Ong J.S., Lew L.C., Hor Y.Y., Liong M.T. (2022). Probiotics: The next dietary strategy against brain aging. Prev. Nutr. Food Sci..

[4] Lavelle A., Sokol H. (2020). Gut microbiota-derived metabolites as key actors in inflammatory bowel disease. Nat. Rev. Gastroenterol. Hepatol..

[5] Xu Y., Zhu Y., Li X., Sun B. (2020). Dynamic balancing of intestinal short-chain fatty acids: The crucial role of bacterial metabolism. Trends Food Sci. Technol..

[6] Li D., Li Y., Yang S., Lu J., Jin X., Wu M. (2022). Diet-gut microbiota-epigenetics in metabolic diseases: From mechanisms to therapeutics. Biomed. Pharmacother..

[7] Catalkaya G., Venema K., Lucini L., Rocchetti G., Delmas D., Daglia M., Filippis A.D., Xiao H., Quiles J.L., Xiao J. (2020). Interaction of dietary polyphenols and gut microbiota: Microbial metabolism of polyphenols, influence on the gut microbiota, and implications on host health. Food Front..

[8] Tan H., Nie S. (2021). Functional hydrocolloids, gut microbiota and health: Picking food additives for personalized nutrition. FEMS Microbiol. Rev..

[9] Yeşilyurt N., Yılmaz B., Ağagündüz D., Capasso R. (2022). Microbiome-based personalized nutrition as a result of the 4.0 technological revolution: A mini literature review. Process Biochem..

[10] Johnson A.J., Vangay P., Al-Ghalith G.A., Hillmann B.M., Ward T.L., Shields-Cutler R.R., Kim A.D., Shmagel A.K., Syed A.N., Students P.M.C. (2019). Daily sampling reveals personalized diet-microbiome associations in humans. Cell Host Microbe.

[11] Kolodziejczyk A.A., Zheng D., Elinav E. (2019). Diet–microbiota interactions and personalized nutrition. Nat. Rev. Microbiol..

[12] Faith J.J., McNulty N.P., Rey F.E., Gordon J.I. (2011). Predicting a human gut microbiota’s response to diet in gnotobiotic mice. Science.

[13] Dey N., Wagner V.E., Blanton L.V., Cheng J., Fontana L., Haque R., Ahmed T., Gordon J.I. (2015). Regulators of gut motility revealed by a gnotobiotic model of diet-microbiome interactions related to travel. Cell.

[14] Gibbons S.M., Gurry T., Lampe J.W., Chakrabarti A., Dam V., Everard A., Goas A., Gross G., Kleerebezem M., Lane J. (2022). Perspective: Leveraging the gut microbiota to predict personalized pesponses to dietary, prebiotic, and probiotic interventions. Adv. Nutr..

[15] Shetty S.A., Kostopoulos I., Geerlings S.Y., Smidt H., de Vos W.M., Belzer C. (2022). Dynamic metabolic interactions and trophic roles of human gut microbes identified using a minimal microbiome exhibiting ecological properties. ISME J..

[16] Kern L., Abdeen S.K., Kolodziejczyk A.A., Elinav E. (2021). Commensal inter-bacterial interactions shaping the microbiota. Curr. Opin. Microbiol..

[17] Beterams A., Arroyo M.C., Paepe K.D., Craemer A.S.D., Elewaut D., Venken K., Wiele T.V.d. (2022). In vitro triple coculture with gut microbiota from spondyloarthritis patients is characterized by inter-individual differences in inflammatory responses. Sci. Rep..

[18] Perdijk O., Baarlen P.V., Fernandez-Gutierrez M.M., Brink E., Schuren F.H.J., Brugman S., Savelkoul H.F.J., Kleerebezem M., Neerven R.J.J.v. (2019). Sialyllactose and galactooligosaccharides promote epithelial barrier functioning and distinctly modulate microbiota composition and short chain fatty acid production in vitro. Front. Immunol..

[19] Maathuis A.J.H., Heuvel E.G.v.d., Schoterman M.H.C., Venema K. (2012). Galacto-oligosaccharides have prebiotic activity in a dynamic *in vitro* colon model using a 13C-labeling technique. J. Nutr..

[20] Vrancken G., Gregory A.C., Huys G.R., Faust K., Raes J. (2019). Synthetic ecology of the human gut microbiota. Nat. Rev. Microbiol..

[21] McDonald J.A.K., Fuentes S., Schroeter K., Heikamp-deJong I., Khursigara C.M., Vos W.M., Allen-Vercoe E. (2015). Simulating distal gut mucosal and luminal communities using packed-column biofilm reactors and an in vitro chemostat model. J. Microbiol. Methods.

[22] Pham V., Mohajeri M. (2018). The application of in vitro human intestinal models on the screening and development of pre-and probiotics. Benef. Microbes.

[23] Vázquez-Castellanos J.F., Biclot A., Vrancken G., Huys G.R., Raes J. (2019). Design of synthetic microbial consortia for gut microbiota modulation. Curr. Opin. Pharmacol..

[24] Elzinga J., van der Oost J., de Vos W.M., Smidt H. (2019). The use of defined microbial communities to model host-microbe interactions in the human gut. Microbiol. Mol. Biol. Rev..

[25] Liaqat I., Naseem S., Eijaz S., Hanif U., Rubab S., Aftab N., Iqbal R. (2021). Impact of interplay between obese gut microbiota and diet in developing obesity in synthetic community mice. J. Oleo Sci..

[26] Venturelli O.S., Carr A.V., Fisher G., Hsu R.H., Lau R., Bowen B.P., Hromada S., Northen T., Arkin A.P. (2018). Deciphering microbial interactions in synthetic human gut microbiome communities. Mol. Syst. Biol..

[27] Rinaldi E., Consonni A., Guidesi E., Elli M., Mantegazza R., Baggi F. (2018). Gut microbiota and probiotics: Novel immune system modulators in myasthenia gravis?. Ann. N. Y. Acad. Sci..

[28] Azad M.A.K., Sarker M., Li T., Yin J. (2018). Probiotic species in the modulation of gut microbiota: An overview. BioMed Res. Int..

[29] Lin L., Du Y., Song J., Wang W., Yang C. (2021). Imaging commensal microbiota and pathogenic bacteria in the gut. Acc. Chem. Res..

[30] Sieow B.F.L., Nurminen T.J., Ling H., Chang M.W. (2019). Meta-omics-and metabolic modeling-assisted deciphering of human microbiota metabolism. Biotechnol. J..

[31] Wang L., Pang X., Zhao J., Jin H., Yang X., Fu S., Cheng S., Li H., Miao C., Man C. (2022). Isolation and characteristics of new phage JK004 and application to control *Cronobacter sakazakii* on material surfaces and powdered infant formula. LWT.

[32] Garcia-Gutierrez E., Cotter P.D. (2021). Relevance of organ (s)-on-a-chip systems to the investigation of food-gut microbiota-host interactions. Crit. Rev. Microbiol..

[33] Nelson M.T., Charbonneau M.R., Coia H.G., Castillo M.J., Holt C., Greenwood E.S., Robinson P.J., Merrill E.A., Lubkowicz D., Mauzy C.A. (2021). Characterization of an engineered live bacterial therapeutic for the treatment of phenylketonuria in a human gut-on-a-chip. Nat. Commun..

[34] Grassart A., Malardé V., Gobaa S., Sartori-Rupp A., Kerns J., Karalis K., Marteyn B., Sansonetti P., Sauvonnet N. (2019). Bioengineered human organ-on-chip reveals intestinal microenvironment and mechanical forces impacting *Shigella* infection. Cell Host Microbe.

[35] Tovaglieri A., Sontheimer-Phelps A., Geirnaert A., Prantil-Baun R., Camacho D.M., Chou D.B., Jalili-Firoozinezhad S., de Wouters T., Kasendra M., Super M. (2019). Species-specific enhancement of enterohemorrhagic *E. coli* pathogenesis mediated by microbiome metabolites. Microbiome.

[36] Marzorati M., Vanhoecke B., Ryck T.D., Sadabad M.S., Pinheiro I., Possemiers S., Abbeele P.V.d., Derycke L., Bracke M., Pieters J. (2014). The HMI™ module: A new tool to study the Host-Microbiota Interaction in the human gastrointestinal tract in vitro. BMC Microbiol..

[37] Jalili-Firoozinezhad S., Gazzaniga F.S., Calamari E.L., Camacho D.M., Fadel C.W., Bein A., Swenor B., Nestor B., Cronce M.J., Tovaglieri A. (2019). A complex human gut microbiome cultured in an anaerobic intestine-on-a-chip. Nat. Biomed. Eng..

[38] Mary P.R., Kapoor M. (2022). Co-culture fermentations suggest cross-feeding among *Bacteroides ovatus* DSMZ 1896, *Lactiplantibacillus plantarum* WCFS1 and *Bifidobacterium adolescentis* DSMZ 20083 for utilizing dietary galactomannans. Food Res. Int..

[39] Thomson P., Medina D.A., Ortúzar V., Gotteland M., Garrido D. (2018). Anti-inflammatory effect of microbial consortia during the utilization of dietary polysaccharides. Food Res. Int..

[40] Egan M., Motherway M.O.C., Kilcoyne M., Kane M., Joshi L., Ventura M., Sinderen D.V. (2014). Cross-feeding by *Bifidobacterium breve* UCC2003 during co-cultivation with *Bifidobacterium bifidum* PRL2010 in a mucin-based medium. BMC Microbiol..

[41] Weiss A.S., Burrichter A.G., Durai Raj A.C., von Strempel A., Meng C., Kleigrewe K., Münch P.C., Rössler L., Huber C., Eisenreich W. (2022). In vitro interaction network of a synthetic gut bacterial community. ISME J..

[42] Hirmas B., Gasaly N., Orellana G., Vega-Sagardía M., Saa P., Gotteland M., Garrido D. (2022). Metabolic modeling and bidirectional culturing of two gut microbes reveal cross-feeding interactions and protective effects on intestinal cells. mSystems.

[43] Liu Y., Heath A.L., Galland B., Rehrer N., Drummond L., Wu X.Y., Bell T.J., Lawley B., Sims I.M., Tannock G.W. (2020). Substrate use prioritization by a coculture of five species of gut bacteria fed mixtures of arabinoxylan, xyloglucan, β-glucan, and pectin. Appl. Environ. Microbiol..

[44] Rivière A., Gagnon M., Weckx S., Roy D., De Vuyst L.J.A., microbiology e. (2015). Mutual cross-feeding interactions between *Bifidobacterium longum* subsp. *longum* NCC2705 and *Eubacterium rectale* ATCC 33656 explain the bifidogenic and butyrogenic effects of arabinoxylan oligosaccharides. Appl. Environ. Microbiol..

[45] Gutiérrez N., Garrido D. (2019). Species deletions from microbiome consortia reveal key metabolic interactions between gut microbes. mSystems.

[46] Petruschke H., Schori C., Canzler S., Riesbeck S., Poehlein A., Daniel R., Frei D., Segessemann T., Zimmerman J., Marinos G. (2021). Discovery of novel community-relevant small proteins in a simplified human intestinal microbiome. Microbiome.

[47] Soto-Martin E.C., Warnke I., Farquharson F.M., Christodoulou M., Horgan G., Derrien M., Faurie J.M., Flint H.J., Duncan S.H., Louis P. (2020). Vitamin biosynthesis by human gut butyrate-producing bacteria and cross-feeding in synthetic microbial communities. mBio.

[48] Rios-Covian D., Gueimonde M., Duncan S.H., Flint H.J., de Los Reyes-Gavilan C.G. (2015). Enhanced butyrate formation by cross-feeding between *Faecalibacterium prausnitzii* and *Bifidobacterium adolescentis*. FEMS Microbiol. Lett..

[49] Chiu L., Bazin T., Truchetet M.E., Schaeverbeke T., Delhaes L., Pradeu T. (2017). Protective microbiota: From localized to long-reaching co-immunity. Front. Immunol..

[50] Magnúsdóttir S., Heinken A., Kutt L., Ravcheev D.A., Bauer E., Noronha A., Greenhalgh K., Jäger C., Baginska J., Wilmes P. (2017). Generation of genome-scale metabolic reconstructions for 773 members of the human gut microbiota. Nat. Biotechnol..

[51] LaSarre B., McCully A.L., Lennon J.T., McKinlay J.B. (2017). Microbial mutualism dynamics governed by dose-dependent toxicity of cross-fed nutrients. ISME J..

[52] Mee M.T., Collins J.J., Church G.M., Wang H.H. (2014). Syntrophic exchange in synthetic microbial communities. Proc. Natl. Acad. Sci. USA.

[53] Guillen M.N., Rosener B., Sayin S., Mitchell A. (2021). Assembling stable syntrophic *Escherichia coli* communities by comprehensively identifying beneficiaries of secreted goods. Cell Syst..

[54] Kong W., Meldgin D.R., Collins J.J., Lu T. (2018). Designing microbial consortia with defined social interactions. Nat. Chem. Biol..

[55] Thommes M., Wang T., Zhao Q., Paschalidis I.C., Segrè D. (2019). Designing metabolic division of labor in microbial communities. mSystems.

[56] LeBlanc J.G., Chain F., Martín R., Bermúdez-Humarán L.G., Courau S., Langella P. (2017). Beneficial effects on host energy metabolism of short-chain fatty acids and vitamins produced by commensal and probiotic bacteria. Microb. Cell Factories.

[57] Kim C.H. (2021). Control of lymphocyte functions by gut microbiota-derived short-chain fatty acids. Cell. Mol. Immunol..

[58] Krautkramer K.A., Fan J., Bäckhed F. (2021). Gut microbial metabolites as multi-kingdom intermediates. Nat. Rev. Microbiol..

[59] Magnúsdóttir S., Thiele I. (2018). Modeling metabolism of the human gut microbiome. Curr. Opin. Biotechnol..

[60] Lin W., Djukovic A., Mathur D., Xavier J.B. (2021). Listening in on the conversation between the human gut microbiome and its host. Curr. Opin. Microbiol..

[61] Gilbert J.A., Lynch S.V. (2019). Community ecology as a framework for human microbiome research. Nat. Med..

[62] Jung D.H., Kim G.Y., Kim I.Y., Seo D.H., Nam Y.D., Kang H., Song Y., Park C.S. (2019). *Bifidobacterium adolescentis* P2P3, a human gut bacterium having strong non-gelatinized resistant starch-degrading activity. J. Microbiol. Biotechnol..

[63] Shetty S.A., Kuipers B., Atashgahi S., Aalvink S., Smidt H., de Vos W.M. (2022). Inter-species metabolic interactions in an in-vitro minimal human gut microbiome of core bacteria. NPJ Biofilms Microbiomes.

[64] Kim G., Bae J.H., Cheon S., Lee D.H., Kim D.H., Lee D., Park S.H., Shim S., Seo J.H., Han N.S. (2022). Prebiotic activities of dextran from *Leuconostoc mesenteroides* SPCL742 analyzed in the aspect of the human gut microbial ecosystem. Food Funct..

[65] Osbelt L., Wende M., Almási É., Derksen E., Muthukumarasamy U., Lesker T.R., Galvez E.J.C., Pils M.C., Schalk E., Chhatwal P. (2021). *Klebsiella oxytoca* causes colonization resistance against multidrug-resistant *K. pneumoniae* in the gut via cooperative carbohydrate competition. Cell Host Microbe.

[66] Sudan S., Flick R., Nong L., Li J. (2021). Potential probiotic *Bacillus subtilis* Isolated from a novel niche exhibits broad range antibacterial activity and causes virulence and metabolic dysregulation in enterotoxic *E. coli*. Microorganisms.

[67] Chen C.C., Lai C.C., Huang H.L., Su Y.T., Chiu Y.H., Toh H.S., Chiang S.R., Chuang Y.C., Lu Y.C., Tang H.J. (2021). Antimicrobial ability and mechanism analysis of *Lactobacillus species* against carbapenemase-producing *Enterobacteriaceae*. J. Microbiol. Immunol. Infect..

[68] Kathayat D., Closs G., Helmy Y.A., Deblais L., Srivastava V., Rajashekara G. (2022). In vitro and in vivo evaluation of *Lacticaseibacillus rhamnosus* GG and *Bifidobacterium lactis* Bb12 against avian pathogenic *Escherichia coli* and identification of novel probiotic-derived bioactive peptides. Probiotics Antimicrob. Proteins.

[69] Chen J., Byun H., Liu R., Jung I.J., Pu Q., Zhu C.Y., Tanchoco E., Alavi S., Degnan P.H., Ma A.T. (2022). A commensal-encoded genotoxin drives restriction of *Vibrio cholerae* colonization and host gut microbiome remodeling. Proc. Natl. Acad. Sci. USA.

[70] Huedo P., Altadill T., Aguiló García M., Sticco M., Perez M., Espadaler-Mazo J. (2022). Probiotic properties of *Bifidobacterium longum* KABP042 and *Pediococcus pentosaceus* KABP041 show potential to counteract functional gastrointestinal disorders in an observational pilot trial in infants. Front. Microbiol..

[71] Piazentin A.C.M., Mendonça C.M.N., Vallejo M., Mussatto S.I., de Souza Oliveira R.P. (2022). Bacteriocin-like inhibitory substances production by *Enterococcus faecium* 135 in co-culture with *Ligilactobacillus salivarius* and *Limosilactobacillus reuteri*. Braz. J. Microbiol..

[72] Boopathi S., Vashisth R., Mohanty A.K., Jia A.Q., Sivakumar N., Alharbi N.S., Khaled J.M., Juliet A., Arockiaraj J. (2022). Investigation of interspecies crosstalk between probiotic *Bacillus subtilis* BR4 and *Pseudomonas aeruginosa* using metabolomics analysis. Microb. Pathog..

[73] Fang K., Jin X., Hong S.H. (2018). Probiotic *Escherichia coli* inhibits biofilm formation of pathogenic *E. coli* via extracellular activity of DegP. Sci. Rep..

[74] Peng M., Tabashsum Z., Patel P., Bernhardt C., Biswas D. (2018). Linoleic acids overproducing Lactobacillus casei limits growth, survival, and virulence of Salmonella Typhimurium and enterohaemorrhagic Escherichia coli. Front. Microbiol..

[75] Divyashree S., Anjali P.G., Somashekaraiah R., Sreenivasa M.Y. (2021). Probiotic properties of *Lactobacillus casei*–MYSRD 108 and *Lactobacillus plantarum*-MYSRD 71 with potential antimicrobial activity against *Salmonella paratyphi*. Biotechnol. Rep..

[76] Hage R.E., Hage J.E., Snini S.P., Ammoun I., Touma J., Rachid R., Mathieu F., Sabatier J.-M., Khattar Z.A., Rayess Y.E. (2022). The Detection of potential native probiotics *Lactobacillus* spp. against *Salmonella* enteritidis, *Salmonella* infantis and *Salmonella* kentucky ST198 of Lebanese chicken origin. Antibiotics.

[77] Mao X., Ma J., Jiao C., Tang N., Zhao X., Wang D., Zhang Y., Ye Z., Xu C., Jiang J. (2021). *Faecalibacterium prausnitzii* attenuates DSS-Induced colitis by inhibiting the colonization and pathogenicity of *Candida albicans*. Mol. Nutr. Food Res..

[78] Hromada S., Qian Y., Jacobson T.B., Clark R.L., Watson L., Safdar N., Amador-Noguez D., Venturelli O.S. (2021). Negative interactions determine *Clostridioides difficile* growth in synthetic human gut communities. Mol. Syst. Biol..

[79] Ghimire S., Roy C., Wongkuna S., Antony L., Maji A., Keena M.C., Foley A., Scaria J. (2020). Identification of *Clostridioides difficile*-inhibiting gut commensals using culturomics, phenotyping, and combinatorial community assembly. mSystems.

[80] Fredua-Agyeman M., Gaisford S. (2019). Assessing inhibitory activity of probiotic culture supernatants against *Pseudomonas aeruginosa*: A comparative methodology between agar diffusion, broth culture and microcalorimetry. World J. Microbiol. Biotechnol..

[81] Khaneghah A.M., Abhari K., Eş I., Soares M.B., Oliveira R.B.A., Hosseini H., Rezaei M., Balthazar C.F., Silva R., Cruz A.G. (2020). Interactions between probiotics and pathogenic microorganisms in hosts and foods: A review. Trends Food Sci. Technol..

[82] Das S., Vishakha K., Banerjee S., Bera T., Mondal S., Ganguli A. (2022). A novel probiotic strain of *Lactobacillus fermentum* TIU19 isolated from Haria beer showing both in vitro antibacterial and antibiofilm properties upon two multi resistant uro-pathogen strains. Curr. Res. Microb. Sci..

[83] Nagarajan V., Peng M., Tabashsum Z., Salaheen S., Padilla J., Biswas D. (2019). Antimicrobial effect and probiotic potential of phage resistant *Lactobacillus plantarum* and its interactions with zoonotic bacterial pathogens. Foods.

[84] Palkovicsné Pézsa N., Kovács D., Rácz B., Farkas O. (2022). Effects of *Bacillus licheniformis* and *Bacillus subtilis* on Gut Barrier Function, Proinflammatory Response, ROS Production and Pathogen Inhibition Properties in IPEC-J2—*Escherichia coli*/*Salmonella Typhimurium* Co-Culture. Microorganisms.

[85] Shin W., Kim H.J. (2018). Intestinal barrier dysfunction orchestrates the onset of inflammatory host–microbiome cross-talk in a human gut inflammation-on-a-chip. Proc. Natl. Acad. Sci. USA.

[86] Shah P., Fritz J.V., Glaab E., Desai M.S., Greenhalgh K., Frachet A., Niegowska M., Estes M., Jäger C., Seguin-Devaux C. (2016). A microfluidics-based in vitro model of the gastrointestinal human–microbe interface. Nat. Commun..

[87] Nelson M.T., Coia H.G., Holt C., Greenwood E.S., Narayanan L., Robinson P.J., Merrill E.A., Litteral V., Goodson M.S., Saldanha R.J. (2022). Evaluation of human performance aiding live synthetically engineered bacteria in a gut-on-a-chip. ACS Biomater. Sci. Eng..

[88] Shin W., Wu A., Massidda M.W., Foster C., Thomas N., Lee D.W., Koh H., Ju Y., Kim J., Kim H.J. (2019). A robust longitudinal co-culture of obligate anaerobic gut microbiome with human intestinal epithelium in an anoxic-oxic interface-on-a-chip. Front. Bioeng. Biotechnol..

[89] Kim H.J., Li H., Collins J.J., Ingber D.E. (2016). Contributions of microbiome and mechanical deformation to intestinal bacterial overgrowth and inflammation in a human gut-on-a-chip. Proc. Natl. Acad. Sci. USA.

[90] Maurer M., Gresnigt M.S., Last A., Wollny T., Berlinghof F., Pospich R., Cseresnyes Z., Medyukhina A., Graf K., Groeger M. (2019). A three-dimensional immunocompetent intestine-on-chip model as in vitro platform for functional and microbial interaction studies. Biomaterials.

[91] Gazzaniga F.S., Camacho D.M., Wu M., Palazzo M.F.S., Dinis A.L.M., Grafton F.N., Cartwright M.J., Super M., Kasper D.L., Ingber D.E. (2021). Harnessing colon chip technology to identify commensal bacteria that promote host tolerance to infection. Front. Cell. Infect. Microbiol..

[92] Moossavi S., Arrieta M.C., Sanati-Nezhad A., Bishehsari F. (2022). Gut-on-chip for ecological and causal human gut microbiome research. Trends Microbiol..

[93] Luna E., Parkar S.G., Kirmiz N., Hartel S., Hearn E., Hossine M., Kurdian A., Flores G.E. (2022). Utilization efficiency of human milk oligosaccharides by human-associated *Akkermansia* is strain dependent. Appl. Environ. Microbiol..

[94] Prete R., Long S.L., Gallardo A.L., Gahan C.G., Corsetti A., Joyce S.A. (2020). Beneficial bile acid metabolism from *Lactobacillus plantarum* of food origin. Sci. Rep..

[95] Lagier J.C., Dubourg G., Million M., Cadoret F., Bilen M., Fenollar F., Levasseur A., Rolain J.M., Fournier P.E., Raoult D. (2018). Culturing the human microbiota and culturomics. Nat. Rev. Microbiol..

[96] Pereira A.C., Cunha M.V. (2020). An effective culturomics approach to study the gut microbiota of mammals. Res. Microbiol..

[97] Rodrigues D.B., Failla M.L. (2021). Intestinal cell models for investigating the uptake, metabolism and absorption of dietary nutrients and bioactive compounds. Curr. Opin. Food Sci..

[98] Liang D., Su W., Tan M. (2022). Advances of microfluidic intestine-on-a-chip for analyzing anti-inflammation of food. Crit. Rev. Food Sci. Nutr..

[99] Ramadan Q., Jing L. (2016). Characterization of tight junction disruption and immune response modulation in a miniaturized Caco-2/U937 coculture-based in vitro model of the human intestinal barrier. Biomed. Microdevices.

[100] Ramadan Q., Jafarpoorchekab H., Huang C., Silacci P., Carrara S., Koklü G., Ghaye J., Ramsden J., Ruffert C., Vergeres G. (2013). NutriChip: Nutrition analysis meets microfluidics. Lab Chip.

